# Mandibular and temporomandibular morphologic characteristics of patients with suspected unilateral condylar hyperplasia: a CT study

**DOI:** 10.1590/2177-6709.25.2.061-068.oar

**Published:** 2020

**Authors:** Diego Fernando López, Juliana Ruiz Botero, Juan M. Muñoz, Rodrigo A. Cardenas-Perilla

**Affiliations:** 1 Universidad del Valle, Departamento de Ortodoncia (Cali, Colombia).; 2 Universidad del Valle, Programa de postgrado en Odontología (Cali, Colombia).; 3 Centro Médico Imbanaco (Cali, Colombia).

**Keywords:** Facial asymmetry, Condylar hyperplasia, Temporomandibular joint, Computed tomography

## Abstract

**Introduction:**

Facial asymmetry associated with unilateral condylar hyperplasia (UCH) is a complicated clinical condition.

**Objective:**

The objective of this study was to describe morphological characteristics of the mandible and the temporomandibular joint in patients with facial asymmetry, using computed tomography and 3D reconstruction.

**Methods:**

A retrospective observational study was performed with patients displaying facial asymmetry evaluated by single photon emission computed tomography (SPECT)/CT analysis, for suspected UCH, between 2015 and 2018. The following variables were compared between the affected side (producing the asymmetry) and the contralateral side (side to where the jaw is deflected): condylar length, condylar medial and lateral pole length, mandibular ramus length, intra-articular spaces, articular eminence height and position of the posterior wall of the glenoid fossa.

**Results:**

Forty-three patients (21 women, mean age: 20.7 ± 7.25 years) with facial asymmetry were included, 19 patients presented right side deviation and 24 patients had left side deviation. Condylar length, lateral pole length, the sum of maximum values and articular eminence height were greater in the affected side (*p*< 0.05). A positive correlation was found between the position of the posterior wall of the glenoid fossa and the articular eminence height in the affected side (r = 0.442).

**Conclusions:**

In patients with suspected UCH, evaluated through CT, craniofacial measurements showed significantly larger condylar length and the condylar sum of maximum values in the affected side. A positive correlation was found between the increased dimensions of the articular eminence and the more posterior position of the glenoid fossa in the affected side.

## RESUMO

**Introdução:**

A assimetria facial associada à hiperplasia condilar unilateral (HCU) é uma condição clínica complexa.

**Objetivo:**

O objetivo desse estudo foi descrever as características morfológicas da mandíbula e da articulação temporomandibular de pacientes com assimetria facial, usando tomografia computadorizada e reconstruções tridimensionais.

**Métodos:**

Esse estudo observacional retrospectivo foi realizado em pacientes com assimetria facial e suspeita de HCU, avaliados por meio da análise de tomografia computadorizada de emissão de fóton único (SPECT)/TC, entre os anos de 2015 e 2018. As seguintes variáveis foram comparadas entre o lado afetado (responsável pela assimetria) e o lado contralateral (lado para onde a arcada estava desviada): comprimento condilar, comprimento dos polos medial e lateral do côndilo, comprimento do ramo mandibular, espaços intra-articulares, altura da eminência articular e posição da parede posterior da fossa glenoide.

**Resultados:**

A amostra constituiu-se de 43 pacientes (21 mulheres, idade média: 20,7 ? 7,25 anos) com assimetria facial, da qual 19 pacientes apresentavam desvio do lado direito e 24, do lado esquerdo. O comprimento condilar, o comprimento do polo lateral, a soma dos valores máximos e a altura da eminência articular foram maiores no lado afetado (*p*< 0,05). Foi encontrada uma correlação positiva entre a posição da parede posterior da fossa glenoide e a altura da eminência articular no lado afetado (r = 0,442).

**Conclusão:**

Em pacientes com suspeita de HCU, avaliados por meio de TC, as medidas craniofaciais mostraram um comprimento condilar e a soma dos valores condilares máximos significativamente maiores no lado afetado. Foi encontrada uma correlação positiva entre as dimensões aumentadas da eminência articular e a posição mais posterior da fossa glenoide no lado afetado.

## INTRODUCTION

The temporomandibular joint (TMJ) is one of the more complex systems of the human body.^1^ TMJ morphologic asymmetries and disorders may be related to a number of different factors, which include inflammatory and degenerative diseases, postural disorders, trauma, premature dental contacts and dentoskeletal asymmetries.^1^ ^-^ ^3^ A common anatomic difference between TMJs of the same subject is clinically perceived as a facial asymmetry, with a reported prevalence of 21 to 85%.^4^ The etiology is not well established, but may be related to genetic or environmental factors appearing during fetal life, childhood or puberty. These factors include Unilateral Condylar Hyperplasia (UCH),^5^ functional disharmonies of the masticatory muscles, dominance of a brain hemisphere,^6^ plagiocephalia, unilateral craniosynostosis, hypoplasic condyle, idiopathic condylar resorption or other unidentified processes. Detection timing contributes to the expression level of the asymmetry.^7^ Changes in position or morphology of the glenoid fossa during growth and functional changes in the mandibular condyle may also have an influence in the development of malocclusions and facial asymmetries as a morphological and functional expression of the alteration, otherwise the occlusion and dental position may modulate their own development based on the continued remodeling of the articular tissues.^8^ ^-^ ^10^ Condylar hyperplasia (CH) is a self-limiting pathologic condition, characterized by progressive condylar overgrowth, but may generate facial deformities compromising the condylar neck and mandibular ramus. The condition is most frequently unilateral, but there have been bilateral cases reported and may be accompanied by pain, occlusal changes and articular disfunctions.^11^ ^,^ ^12^ Condylar asymmetry may be due to overgrowth in vertical direction, a condition known as hemimandibular hyperplasia, or horizontal, known as hemimandibular elongation, or an hybrid form of them.^13^ ^,^ ^14^ Each type has different clinical and radiographic morphological characteristics.^15^ Recent technological advances in craniofacial imaging, particularly 3D computed tomography, provide volumetric information and virtual 3D reconstructions that are valuable to more precisely visualize the TMJ, as well as to obtain differential diagnosis of these asymmetries.^16^ ^,^ ^17^ The objective of this study was to describe morphological characteristics of the mandible and the temporomandibular joint in patients with facial asymmetry using computed tomography (CT) and 3D reconstruction. 

## MATERIAL AND METHODS

This study was approved by the *Comité de Ética en Investigación del Centro Médico Imbanaco* (CEI-285) and conducted according to the principles of the Declaration of Helsinki. A retrospective observational study was performed in 43 patients who had been sent to a high complexity nuclear medicine center for a single photon emission computed tomography (SPECT/CT) test, because they had progressive facial asymmetry and a positive association of extraoral and intraoral clinical findings with radiographic signs, leading to suspicion of a UCH, between January 2015 and December 2018. Mean age was 20.7 ± 7.25 year (range:11-44 years) and 21 were women. 

The institutional registry of nuclear medicine department was reviewed to identify patients with a SPECT/CT study due to a preliminary clinical diagnosis of facial asymmetry and clinically suspected UCH. Patients with antecedents of TMJ surgery, orthognathic surgery, craniofacial trauma or syndromic dentofacial anomalies were excluded. 

The CT cranial images were obtained from a PET/CT Biograph mCT20 (Siemens, Erlangen, Germany), without contrast medium and under the following parameters: section width: 1.5 mm, pitch 1.0 using Care Dose (dose adjusted to patient’s weight), and a 512 x 512 cubic matrix of isotropic voxel (size: 0,58 x 0,58 x 0,87 mm) that prevents distortion of the image in the different views. The CT images were reconstructed with a B26f homogenous medium filter. All the patients were positioned with head restriction, to avoid movement artifacts and to permit the image fusion with SPECT images. The set of DICOM images was processed in a computer workstation using the Osirix software (Pixmeo, Bernex, Switzerland) v. 7.5.1. Linear measurements in sagittal, axial and coronal directions were obtained. The variables and detailed methodology are described in Table 1. All variables were compared between the affected side (producing the asymmetry) and the contralateral side (side with mandibular deviation).

**Table 1:** Description of variables measured from CT images and 3D reconstruction.

Variable	Description
Condylar length	In the sagittal view, it was traced a line parallel to the tangent to the posterior edge of the mandibular ramus, extended from the most superior point of the condyle to a perpendicular line passing through the most inferior point of the mandibular notch (Fig. 1A). This length was obtained from a corrected plane image over the long axis of the mandibular ramus
Mandibular ramus length	In the sagittal view, it was traced a line, parallel to the tangent to the posterior edge of the mandibular ramus, extended from the most inferior point of the mandibular notch to a perpendicular line extended from the internal mandibular angle (Fig. 1A)
Intra-articular anterior space	In sagittal view, on the TMJ (center of the condyle, set in the coronal plane - orthogonal plane), it was traced a line from the most anterior edge of the condyle to the anterior wall of the glenoid fossa (Fig. 1B)
Intra-articular upper space	In sagittal view, on the TMJ (center of the condyle, set in the coronal plane - orthogonal plane), it was traced a line from the most superior edge of the condyle to the most superior point of glenoid fossa (Fig. 1B)
Intra-articular posterior space	In sagittal view, on the TMJ (center of the condyle, set in the coronal plane - orthogonal plane), it was traced a line from the most posterior edge of the condyle to the posterior wall of glenoid fossa (Fig. 1B)
Glenoid fossa posterior wall position	Taking as a reference the center of sella turcica in sagittal and axial views, on the axial plane, a perpendicular line was draw over the facial midline. The distance between this line and the posterior wall of the glenoid fossa was measure (Fig. 2A). A greater value indicates a more posterior position and a lower value, an anterior position
Condylar anteroposterior pole length	In the axial view, it was traced a line from the most anterior edge of cortical bone to the most posterior edge of condylar cortical bone (Fig. 2B). This image was obtained in an orthogonal plane
Condylar mediolateral pole length	In the axial view, it was traced a line from the most anterior edge of condyle proximal cortical to the most anterior edge of condylar distal cortical (Fig. 2B). This image was obtained in an orthogonal plane
Sum of maximum values	Obtained by adding the values of the condylar length with the condylar anteroposterior pole length and the condylar lateral pole length, representing the condylar size in its maximum values
Articular eminence height	In the frontal view, taking as reference a line crossing the anatomic point sella turcica (parallel), it was traced a perpendicular from the most superior medial cortical edge of the articular eminence to the reference line (Fig. 3). A lower value represents a more superior position of the articular eminence height and a higher value, the opposite
Deviation from the symphysis midpoint	Distance from menton point to a line extended to the lower third, projected from the middle part of crista galli apophysis, perpendicular to bizygomatic line in the reconstruction 3-D image of bone tissues (Fig. 4)
Laterognathia (side of mandibular deviation)	Visual qualitative variable indicating direction of the mandibular deviation (right/left). Observed in the reconstruction 3D image of bone tissues (Fig. 4)

**Figure 1:**
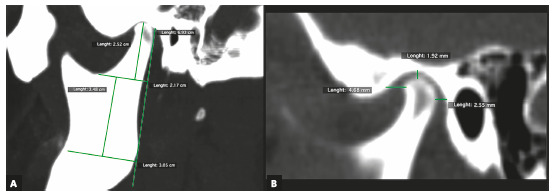
Sagittal TMJ view. A) Measurement of condylar length and mandibular ramus length. B) Intra-articular anterior, upper and posterior spaces.

**Figure 2:**
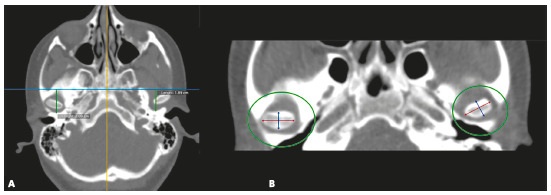
A) Measurement of glenoid fossa posterior wall position. B) Axial view, condylar medial and lateral pole length.

**Figure 3:**
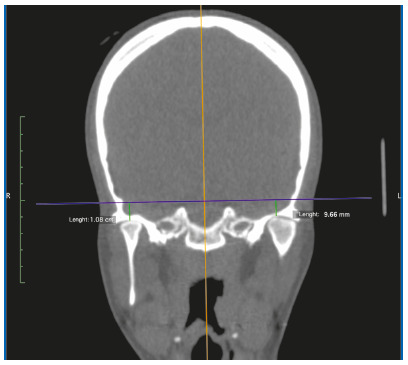
Frontal view: measurement of articular eminence height.

**Figure 4:**
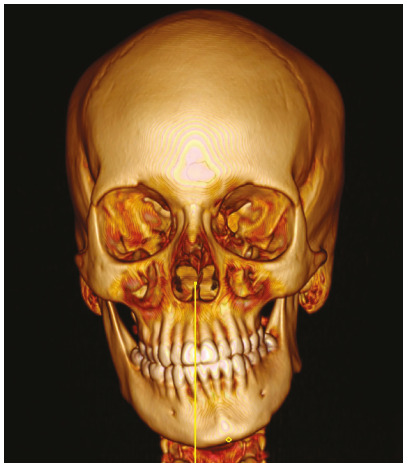
3-D reconstruction of bone tissues, used to measure the distance from the midpoint of mandibular symphysis to sagittal midplane (magnitude of deviation).

## METHODS ERROR

The variables were obtained and processed by one expert operator. The measurements were made twice at more than 1-week intervals, without any knowledge of the previous measurements, with the objective of measuring the intraobserver reliability, and it was estimated using the intraclass correlation coefficient (ICC) (Table 2).

**Table 2:** Intraobserver reliability.

Variable	ICC	ICC
	Right side	Left side
Condylar length	0.963	0.985
Ramus length	0.966	0.982
Anteroposterior pole length	0.976	0.944
Mediolateral pole length	0.955	0.962
Glenoid fossa posterior wall	0.997	0.998
Eminence medial height	0.989	0.991
Intra-articular antero-superior space	0.762	0.623
Intra-articular medial space	0.904	0.899
Intra-articular upper posterior space	0.905	0.870

ICC: Intraclass correlation coefficient.

## STATISTICAL ANALYSIS

All analysis were conducted using Stata 13^®^ (StataCorp, College Station, TX, USA). Data were explored for normality using Shapiro-Wilk test. The difference between sides was expressed in mm, respecting the contralateral side (Δ= [Affected-Contralateral]). To compare if the differences were statistically different than 0, the paired t-test or Wilcoxon test was used according to the normality assumption. The correlation between variables was calculated using Spearman Coefficient; *p*< 0.05 was considered statistically significant.

## RESULTS

A total of 19 cases with right side deviation and 24 with left side deviation were analyzed. The average mandibular deviation was higher in cases with left side deviation (left deviation: 6.2 ± 3.8 mm, right deviation: 4.5 ± 2.9 mm), however, this difference was not statistically significant (*p =* 0.131). 

The condylar and articular morphologic differences between the affected side and the contralateral side are described in Table 3. No statistically significant differences were found between the sides regarding the anteroposterior pole, mandibular ramus length, intra-articular medial and posterior spaces, and posterior wall of the glenoid fossa (*p*> 0.05). 

**Table 3:** Comparison of morphologic measurements by side (affected / contralateral) and deviation side, in millimeters.

Variables	Affected side	Contralateral side	Difference	P value	Δ>0 n (%)
Condylar length					
Mean ± SD	22.8±3.1	20.1±2.9	2.7±2.4	0.000***	36 (83.7%)
(Range)	(16.5; 30.6)	(14.9; 26.5)	(-1.4; 8.5)		
Anteroposterior pole					
Mean ± SD	7.9±1.2	7.6±1.3	0.3±1.1	0.097*	-
(Range)	(5.9;11.1)	(5.3;10.6)	(-2.0;2.9)		
Mediolateral pole					
Mean ± SD	17.3±2.3	16.7±2.6	0.6±1.9	0.039**	26 (60.5%)
(Range)	(12.8;22.0)	(11.5;23.3)	(-3.7; 5.5)		
Sum of maximum values					
Mean ± SD	48.1±4.3	44.4±4.7	3.7±3.8	0.000***	34 (79.1%)
(Range)	(38.9; 57.3)	(35.5;54.8)	(-4.0; 12.0)		
Mandibular ramus					
Mean ± SD	34.4±4.1	34.21±4.4	0.2±3.0	0.668	-
(Range)	(24.3;42.1)	(24;43.4)	(-5.6;6.6)		
Eminence height					
Mean ± SD	14.0±4.2	14.5±4.1	-0.5±1.6	0.029**	26 (60.5%)
(Range)	(4.7; 24.4)	(5.0; 23.9)	(-3.6; 2.8)		
Glenoid fossa posterior wall					
Mean ± SD	19.9±5.9	19.3±6.3	0.6±3.9	0.308	28 (66.7%)
(Range)	(10.5; 31.6)	(7.2; 31.5)	(-8.7; 9.5)		
Intra-articular anterior space					
Mean ± SD	2.3±0.8	2.4±0.8	-0.1±1.0	0.551	-
(Range)	(1.0; 4.2)	(1.2; 4.7)	(-2.5; 1.4)		
Intra-articular medial space					
Mean ± SD	1.8±0.7	2.0±0.8	-0.2±0.7	0.104	-
(Range)	(0.6; 3.5)	(0.6; 4.0)	(-2.0; 1.6)		
Intra-articular posterior space					
Mean ± SD	2.8±1.1	2.8±1.0	-0.0±1.0	0.642	-
(Range)	(1.2; 5.5)	(0.9; 5.6)	(-2.1; 1.8)		

SD: Standard deviation; * p<0.10; ** p<0.05; ***p<0.001; Δ= [affected-contralateral].

Condylar length (*p*< 0.001) and lateral pole length (*p*< 0.05) were greater on the affected side. The distance from the upper edge of the eminence to the reference line had lower values on the affected side, suggesting a higher superior projection of the joint eminence on the affected side (*p*< 0.05). Approximately 84% of the patients presented greater condylar length on the affected side and 79%, greater condylar size according to the sum of maximum values. In 61% of the cases, a higher length of the lateral pole and a greater eminence height on the affected side were observed. Although, the differences in mm of the position of the posterior wall of the glenoid fossa between the sides were not statistically significant, 67% of the cases had a greater posterior projection of the glenoid cavity on the affected side (Table 3). A positive Spearman correlation coefficient was obtained between the position of the posterior wall of the glenoid cavity and the height of the articular eminence (r = 0.442, *p*= 0.003).

## DISCUSSION

The morphology of TMJ structures as well as the spatial disposition, remodeling during growth and adaptability to occlusal functional demands has been a constant challenge in the diagnostic and multidisciplinary treatment of malocclusions and asymmetries that compromise the middle and lower facial thirds^17^. The principal outcome of this study suggests that there are changes in size and anatomic configuration of the mandibular condyle as well as structural adaptations in the glenoid fossa when it is affected by a process of hyperplasia that causes facial asymmetry. The diagnostic implications of these morphological changes demand from both clinicians in the areas of orthodontics and maxillofacial surgery. The knowledge and understanding of the craniofacial characteristics that distinguish the UCH from other entities that also produce facial asymmetry are relevant for an adequate diagnosis and therapeutic approach. 

Previous data indicate that most cases associated with UCH are detected in young adults, but the range is initiated in preadolescence, in agreement with the reports of Wolford et al.^12^ UCH is usually diagnosed in its active form between 11 and 25 years of age, therefore patients with growth and development or with residual growth are always involved in the diagnostic process. Although there may be active cases in the third and fourth decade of life, the most usual is to find in this stage the disease sequel (anatomical, occlusal, functional and aesthetic alterations).^11^

In patients with facial asymmetry, previous studies have described that the predisposition to develop TMJ internal derangements could be attributed to an adaptation process of skeletal and dentofacial structures to the mandibular displacement and the functional demands placed by the occlusal configuration.^18^ The articular disorders have also been related to a reduced length of the mandibular ramus and body, reduced length in anterior and posterior cranial base and articular disk compression.^19^ Additionally, the difference in height of the condyles could be a compensatory response to an abnormal remodeling of the glenoid fossa due to a pathological condition. 

Disorders such as unilateral anterior displacement of the articular disk during growth and development are also likely to reduce the condylar length of the affected side, triggering asymmetry.^20^ Habib et al.^21^ and Kurita et al.^22^ showed that changes in TMJ function alter the volume of the condylar head. Changes in the volume of soft tissues are related to other disorders, such as metabolic bone pathologies, including CH that may involve changes in condylar anatomy.^11^ ^,^ ^12^ ^,^ ^23^

Goulart et al.^24^ evaluated the condylar volume in patients with UCH compared to Class III malocclusion patients. They concluded that in UCH patients the affected condyle presented a higher volume than the contralateral condyle, while in the Class III group the condylar volume was equal for both sides. These results are similar to this study, with a higher sum of maximum values identified in the hyperplasic side. 

In this study, condylar length and condylar lateral pole length were higher in the affected side. These findings are similar to those reported by Goto and Langenbach,^25^ who evaluated 40 asymmetric patients finding that the condylar size in the deviated side was significantly larger, compared with a control group. Velasquez et al.^26^ analyzed 40 cone-beam CT images from patients with mandibular lateral deviation, evaluating craniofacial morphology. They found that mandibular body length and condylar size were larger in the deviation side, compared to the contralateral side. 

On the other hand, Ishizaki et al.^18^ analyzed 116 bi-dimensional diagnostic images from patients with mandibular lateral deviation, relating their occlusal, functional and morphologic characteristics. They reported that the mandible presented rotation, together with condylar displacement towards the contralateral side. The condyle affected by the mandibular displacement compressed the disk against the glenoid fossa during masticatory function or parafunctional activities. Their results are contrary to the present study, as it was not found intra-articular space reduction, suggesting condylar compression in the contralateral side. 

Regarding differences due to malocclusions, Katsavrias and Halazonetis^27^ studied the articular structures in Class II and Class III patients, concluding that in Class III cases the condylar sagittal position was intermediate, while in Class II division 2 cases, the vertical position of the condyle was more posterior to the glenoid fossa. They stated that condylar anatomic characteristics may be changed by continuous structural remodeling of the TMJ and symmetric condyles may be found in different kinds of malocclusion. In the present study the malocclusion class was not a variable considered.

According to the results of the present study, when the articular eminence height is increased in the affected side, there is also an increment in the posterior position of the glenoid fossa. If this is correlated to the increment in the sum of maximum values in the affected side, it may be interpreted as expression of the glenoid fossa remodeling to increase the space, when the condylar size increased in UCH situations. The same findings are reported by Huang et al.,^28^ correlating depth of the glenoid fossa with articular eminence height. The results suggest that changes in the position of glenoid fossa occur during normal growth or in pathologic conditions such as UCH, and they may have an influence on the development of a malocclusion and facial asymmetry, as morphologic and functional expressions of the alteration. On the other hand, the occlusion and dental position may modulate or adapt to the continuous remodeling of the articular morphology.^8^ ^-^ ^10^

A limitation of this study is that it was not possible to correlate the anatomic changes in TMJ structures with signs and symptoms of temporomandibular disfunction. Therefore, it is not known if morphologic changes in UCH patients may increase the actual risk of temporomandibular disorders either in the affected side or in its contralateral side, or if TMD may facilitate the degree of hyperplastic expression. It is suggested to perform in future studies an analysis between transversal changes in the three facial thirds and metabolic activity of condyles evaluated by SPECT, as well as histopathologic findings in patients after high condylectomyis used to treat active hyperplasia.

## CONCLUSIONS

In patients with suspected UCH, evaluated through CT, craniofacial measurements showed significantly larger condylar length and the condylar sum of maximum values in the affected side. A positive correlation was found between the increased dimensions of the articular eminence and the more posterior position of the glenoid fossa in the affected side. No significant differences were detected for articular spaces or mandibular ramus height in the affected side, compared to the normal side. 
